# Proteomic Analysis of Sauvignon Blanc Grape Skin, Pulp and Seed and Relative Quantification of Pathogenesis-Related Proteins

**DOI:** 10.1371/journal.pone.0130132

**Published:** 2015-06-15

**Authors:** Bin Tian, Roland Harrison, James Morton, Santanu Deb-Choudhury

**Affiliations:** 1 Department of Wine, Food and Molecular Biosciences, Lincoln University, Lincoln, 7647, Canterbury, New Zealand; 2 Agresearch, Lincoln Research Centre, Christchurch, 8140, Canterbury, New Zealand; Universita degli Studi di Siena, ITALY

## Abstract

Thaumatin-like proteins (TLPs) and chitinases are the main constituents of so-called protein hazes which can form in finished white wine and which is a great concern of winemakers. These soluble pathogenesis-related (PR) proteins are extracted from grape berries. However, their distribution in different grape tissues is not well documented. In this study, proteins were first separately extracted from the skin, pulp and seed of Sauvignon Blanc grapes, followed by trypsin digestion and analysis by liquid chromatography-electrospray ionization-tandem mass spectrometry (LC-ESI-MS/MS). Proteins identified included 75 proteins from Sauvignon Blanc grape skin, 63 from grape pulp and 35 from grape seed, mostly functionally classified as associated with metabolism and energy. Some were present exclusively in specific grape tissues; for example, proteins involved in photosynthesis were only detected in grape skin and proteins found in alcoholic fermentation were only detected in grape pulp. Moreover, proteins identified in grape seed were less diverse than those identified in grape skin and pulp. TLPs and chitinases were identified in both Sauvignon Blanc grape skin and pulp, but not in the seed. To relatively quantify the PR proteins, the protein extracts of grape tissues were seperated by HPLC first and then analysed by SDS-PAGE. The results showed that the protein fractions eluted at 9.3 min and 19.2 min under the chromatographic conditions of this study confirmed that these corresponded to TLPs and chitinases seperately. Thus, the relative quantification of TLPs and chitinases in protein extracts was carried out by comparing the area of corresponding peaks against the area of a thamautin standard. The results presented in this study clearly demonstrated the distribution of haze-forming PR proteins in grape berries, and the relative quantification of TLPs and chitinases could be applied in fast tracking of changes in PR proteins during grape growth and determination of PR proteins in berries at harvest.

## Introduction

Protein stabilization of white wine is of great concern to winemakers as denaturation of proteins in wine may cause haze formation, which is usually considered a wine fault. Pathogenesis-related (PR) proteins originally derived from grape berries are the major soluble proteins remaining in finished wine and they are mainly responsible for haze formation [1,2]. Pathogenesis-related proteins are a group of plant proteins induced in pathological or related situations [3]. They were first discovered in tobacco as a result of a hypersensitive reaction to tobacco mosaic virus (TMV) [4]. Pathogenesis-related proteins are typically acidic, of low molecular mass and highly resistant to proteolytic degradation and to low pH values. On the basis of similarities in amino acid sequences, serological relationship, and/or enzymatic or biological activity, eleven families have been recognized and classified for tobacco and tomato [5]. Some of these PR protein family members have also been found in grapevine. The two prominent soluble proteins accumulated in grapes during ripening have been identified as chitinases (PR-3 family) and thaumatin-like proteins (PR-5 family) [6,7]. However, in early studies, the β-1,3-glucanases (PR-2 family), a potential indicator of pathogen attack, were not found in grape juice and/or berry extracts [7–10]. With the accomplishment of grapevine genome sequencing programmes in 2007 [11,12] and the development of technology in protein analysis, proteomic analysis of grapevine has significantly improved knowledge of grape proteins and produced a better understanding of their characteristics [13]. These have identified other PR protein family members found in grapevine, such as osmotins (PR-5 family), β-1,3-glucanases (PR-2 family) and the PR-10 proteins [14–16].

Thaumatin-like proteins (TLPs) and chitinases are the two predominent PR protein families present in finished white wine [2,10,17] and they are usually removed by fining with bentonite, a clay material that has a strong affinity for proteins and other larger molecules [18]. However, the addition of bentonite may result in the loss of wine volume (5–20%) as lees and remove important aroma and flavour compounds [19,20]. Recent study showed that bentonite requirement to achieve wine protein stability is strongly correlated with concentration of PR proteins in wine, and specifically has a positive linear correlation with the concentration of chitinases [21]. Thus, a lower concentration of PR proteins in juice and wine, in particular the concentration of chitinases, could reduce the bentonite usage required in white wine protein stabilization. Since both TLPs and chitinases found in wine are derived from grape berries, the distribution and quantification of them in grape berries could be of great interest for winemakers to potentially reduce their concentrations in juice through managing the extraction during juice processing. Deytieux and co-workers have observed that the TLPs and chitinases are present in the skin of *Vitis vinifera* L. cv. Cabernet sauvignon, and their concentrations in grape skin increase during ripening [14]. A recent study on the effects of water stress on grapes [22] also shows the presence of chitinases in pericarp (skin and pulp). Proteomic studies since the completion of grapevine genome sequencing in 2007 have investigated the diversity of PR proteins [23] and protein changes during ripening [24], but there is little reported on the distribution and quantification of PR proteins in specific grape tissues, especially with focus on white wine haze formation related TLPs and chitinases. Therefore, in this study, the liquid chromatography-electrospray ionization-tandem mass spectrometry (LC-ESI-MS/MS) was carried out to investigate the distrubition of PR proteins in different grape tissues and provide some initial assessment towards quantification. The protein extracts of grape tissue were also analysed by high performance liquid chromatography (HPLC) and sodium dodecyl sulphate polyacrylamide gel electrophoresis (SDS-PAGE) to obtain the relative quantification of TLPs and chitinases in grape tissues.

## Materials and Methods

### Sauvignon Blanc grapes and protein extraction

Sauvignon Blanc grapes were collected from the Dillons Point vineyard (with permission granted from the owner of vineyard) in Marlborough, New Zealand (41°30'52.9"S, 174°01'15.5"E) at harvest in 2012. The grape skin was obtained by hand-peeling 20 frozen grape berries and the pulp was accordingly obtained by removing the seeds. Protein extraction from the grape skin, pulp and seed was then carried out according to a method optimised for plant tissues rich in phenolics [25]. Approximately 2 g of skin, 2 g of pulp and 0.5 g of seed were ground to a fine powder in liquid nitrogen with mortar and pestle. The powder was vortexed in 5 mL of sucrose buffer (0.7 M sucrose, 0.5 M Tris-HCl pH 7.5, 50 mM EDTA, 0.1 M potassium chloride, 2 mM PMSF, 2% 2-ME and 1% PVP) and incubated for 30 min at 4°C. An equal volume of 1 M Tris-saturated phenol (pH 7.9) was added. The mixture was stored at -20°C for 30 min with vortexing every 10 min. The phases were separated by centrifugation (for 30 min at 0°C and at 3210 g). The upper phenol phase was collected and re-extracted twice with an equal volume of sucrose buffer. From 5 mL initially collected of the phenol phase, 2 mL was recovered after two re-extractions. Five volumes of 0.1 M w/v ammonium acetate in cold methanol were added to the phenol phase followed by incubation at -20°C overnight to precipitate proteins. The pellet was washed three times with 5 mL of cold 0.1 M ammonium acetate/methanol (w/v) and once with 5 mL of cold acetone before the trypsin digestion treatment. The protein pellet was further utilised either for the LC-ESI-MS/MS analysis or for the HPLC analysis.

### Trypsin digestion and LC-ESI-MS/MS

The washed protein pellet was dissolved in 100 μL 50 mM ammonium bicarbonate by sonication for 5 minutes. The dissolved proteins were then reduced with 50 mM tris-(2-carboxyethyl) phosphine (TCEP), alkylated with 360 mM acrylamide and finally digested with sequencing grade trypsin (Promega). Nanoflow LC-MS/MS was performed on a Nano-Advance (Bruker) HPLC. Samples were loaded onto a C_18_ trap column and then switched in-line with an analytical column (Bruker, 0.1 x 150 mm Magic C_18_ AQ 3.0μm, 200Å). Elution was performed at 0.8 μL/min, using a tailored gradient from 0%-35% acetonitrile (with 0.1% formic acid) over 60 minutes and then from 35%-45% acetonitrile (with 0.1% formic acid) in 10 minutes. The column outlet was directly interfaced to an amaZon Speed ETD (Bruker) mass spectrometer. Automated information dependent acquisition (IDA) was performed using Hystar PP 3.2.44.0 software, with a MS survey scan over the range m/z 350–1200 followed by three MS/MS spectra from 50–3000 m/z acquired during each cycle of 30 ms duration.

### Analysis of MS/MS data

After each LC-MS/MS run, peak lists were queried against *Vitis vinifera* sequences in the Uniprot database using the Mascot search engine (v2.2.03, Matrix Science) maintained on an in-house server. The following Mascot search parameters were used: ‘semitrypsin’ was selected as the proteolytic enzyme with two missed cleavages permitted; error tolerance was set to 0.3 Da for MS and 0.6 Da for MS/MS. Search results were compiled and analysed using ProteinScape 3.1.0 (Bruker) using the ProteinExtractor function. Acceptance thresholds for peptide and protein scores were set at 20 and 80, respectively. The identification score for at least one peptide used for protein identification was calculated by the search engine. Results assessed as being true matches were used for further analysis.

### Assignment of identified proteins to functional classes

Identified proteins were assigned a Gene Ontology (GO) term according to their molecular function. Protein NCBI accession numbers were used to perform batch retrieval on the Protein Information Resource website (http://pir.georgetown.edu/), and grouped into functional categories using the ‘GO-MIPS funcat conversion table’ (http://www.geneontology.org/external2go/mips2go) set up at the Munich Information Center for Protein Sequences (MIPS Institute) [26]. In some cases, where the GO term assigned to the protein appeared too broad, proteins were assigned to MIPS funcats (http://www.mips.gsf.de/projects/funcat) according to their role described in the literature.

### HPLC analysis of protein extracts

The washed protein pellet was dissolved in 1 mL 7 M urea. Protein extracts (50 μL) of grape tissues, Sauvignon Blanc juice (50 μL), and purified thaumatin-like proteins (TLPs) and chitinases which were prepared using the two step purification method decribed by Van Sluyter et al. [27], were loaded at 1 mL/min onto a C8 guard column (4.6x5 mm, Vydac 208GK54) which was equilibrated using a mixture of 83% (v/v) solvent B [0.1% trifluoroacetic acid (TFA) in 8% acetonitrile] and 17% solvent A [80% acetonitrile, 0.1% (v/v) TFA] at 35°C at a flow rate of I ml/min. HPLC analysis was performed using a C8 column (4.6x250 mm, Vydac 208TP54) equilibrated with the above solvent mix. A gradient elution of the proteins was performed; 17% solvent A to 49% solvent A in the first 7 min, from 49 to 57% in 7 to 15 min, from 57 to 65% in 15 to 16 min, from 65 to 81% in 16 to 30 min, and then held at 81% for 5 min before re-equilibrating the column in the starting conditions for an additional 6 min [28]. Elution was monitored using wavelengths at 210, 220, 260, 280, and 320 nm. Sauvignon Blanc juice proteins eluted at 9.2 min and 19.2 min were assigned to TLPs and chitinases respectively according to the previous studies [10,29,30]. For the protein extracts of grape tissues, since there was only a single peak at 19.2 min which was assigned to chitinases without further investigation. However, there were two possible peaks, eluting at 9.3 min and 10.6 min respectively, which might correspond to TLPs in this study. Thus, proteins from these two peaks were collected and labelled as fraction 1 (F1) and fraction 2 (F2) accordingly and further investigated by sodium dodecyl sulphate polyacrylamide gel electrophoresis (SDS-PAGE). Relative quantification of TLPs and chitinases in the protein extracts using HPLC was carried out by comparison of the corresponding peak areas against the area of a commercial thaumatin protein from *Thaumatococcus daniellii* (Sigma-Aldrich, New Zealand) used as a standard, and thus the protein concentration was expressed as thaumatin equivalent.

### Protein composition analysis by SDS-PAGE

Fractions F1 and F2 of protein extracts and TLPs collected from Sauvignon Blanc juice were freeze-dried and re-dissolved in 30 μL of Tris-HCl buffer (pH 7.5). Aliquots of these concentrates (15 μL) were mixed with 5 μL of the NuPAGE LDS sample buffer (Novex, Life Technologies, US) and then denatured at 70°C for 10 minutes. Denatured protein samples were then loaded onto the NuPAGE Bis-Tris Mini Gel (Novex, Life Technologies, US). Electrophoresis was run in MOPS SDS running buffer with constant voltage mode (200 V) at room temperature for 50 min. The gel was stained using SimplyBlue SafeStain (Novex, Life Technologies, US) according to the manufacturer’s protocol.

## Results and Discussion

### Identification of PR proteins in grape tissues

Proteins extract from skin, pulp and seed tissues of Sauvignon Blanc grapes and analysed by LC-MS and are shown in Table 1. Hypothetical proteins, uncharacterised proteins and unnamed proteins are excluded from this table. Genome sequencing has greatly enhanced the analysis of the proteome of *Vitis vinifera* [12]. In this study, over 100 proteins were identified in specific Sauvignon Blanc grape tissues. This direct injection method provides rapid identification of the major proteins extracted from grape tissues, but high intensity peaks from liquid chromatography separation may eclipse lower intensity peaks due to the lack of pre-fractionation, so proteins with low concentration in protein mixture may be less easily encountered and identified in whole tissue proteomic studies. Among the identified proteins, 38 annotated proteins were present in both grape skin and pulp, 15 annotated proteins present in both grape pulp and seed, and 11 annotated proteins present in both grape skin and seed. However, some of the identified proteins were exclusively present in specific grape tissues: proteins involved in photosynthesis such as chlorophyll a-b binding protein (gi|225447576) and photosystem II 44 kDa protein (gi|91983988), were only identified in grape skin; and proteins found in alcoholic fermentation such as alcohol dehydrogenase 7 (gi|7264742) and pyruvate decarboxylase (gi|10732644), were only identified in grape pulp.

**Table 1:** Identified proteins and their distribution in Sauvignon Blanc grapes using LC-MS/MS.

Identified proteins	NCBI database accession	MW [kDa]	pI	skin	pulp	seed
**01 Metabolism**						
*01*.*01 Amino acid metabolism*						
5-methyltetrahydropteroyltriglutamate-homocysteine methyltransferase	225439223	84.90	6.08	-	-	+
serine hydroxymethyltransferase, mitochondrial	225429452	57.10	8.94	+	-	-
serine hydroxymethyltransferase 1	225433510	51.90	7.80	-	+	-
*01*.*05 C-compound and carbohydrate metabolism*						
1,3 beta glucanase	6273716	13.40	6.11	+	-	-
2,3-bisphosphoglycerate-independent phosphoglycerate mutase isoform 2	359480976	60.20	5.63	+	-	+
5'-methylthioadenosine/S-adenosylhomocysteine nucleosidase 1 isoform 2	359475059	28.30	4.59	+	-	-
adenosylhomocysteinase-like isoform 1	225456806	53.00	5.71	+	+	-
alpha-1,4 glucan phosphorylase L isozyme, chloroplastic/amyloplastic-like	359489019	111.30	5.05	+	+	-
beta-glucosidase 42-like	359495874	55.20	5.09	+	-	-
chitinase 5-like	225434076	43.40	6.51	+	+	-
class IV endochitinase	2306811	27.20	5.31	+	+	-
endochitinase-like	359497495	21.20	8.94	-	-	+
isocitrate lyase-like	225447308	64.60	7.07	-	-	+
phosphoglycerate kinase, chloroplastic	359494603	40.90	9.54	+	-	-
phosphoglycerate kinase, cytosolic	225464999	42.40	6.31	+	+	-
probable galactinol—sucrose galactosyltransferase 2	225441787	84.80	5.43	+	+	-
Putative 2–3 biphosphoglycerate independant phosphoglycerate mutase	239056191	61.00	5.57	-	+	-
pyrophosphate—fructose 6-phosphate 1-phosphotransferase subunit alpha-like	225457674	67.30	8.78	+	+	-
ribulose bisphosphate carboxylase/oxygenase activase 2, chloroplastic isoform 2	359478916	48.60	5.78	+	-	-
sedoheptulose-1,7-bisphosphatase, chloroplastic	225466690	42.50	5.92	+	-	-
sucrose synthase 2	225437428	92.40	5.69	-	+	+
UDP-glucuronic acid decarboxylase 1	225449563	38.80	6.53	+	+	+
UTP—glucose-1-phosphate uridylyltransferase isoform 2	359476943	50.20	6.41	+	+	-
*01*.*06 Lipid*, *fatty acid and isoprenoid metabolism*						
9,10[9',10']carotenoid cleavage dioxygenase	61654494	61.10	6.04	+	-	-
acyl-[acyl-carrier-protein] desaturase, chloroplastic-like	359496940	45.00	7.87	+	-	-
annexin D1	225459318	35.20	7.82	+	+	-
lipid transfer protein isoform 4	28194086	11.70	10.40	+	-	-
lipoxygenase	268636245	101.60	6.06	+	-	-
non-specific lipid-transfer protein	225439679	11.60	10.52	+	-	-
non-specific lipid-transfer protein 2-like	359490972	9.70	9.18	+	-	-
non-specific lipid-transfer protein A-like	225446753	11.90	9.96	-	-	+
phospholipase D alpha	84620126	91.70	5.52	+	+	-
probable acetyl-CoA acetyltransferase, cytosolic 2	225447510	41.10	6.00	+	+	+
probable acetyl-CoA acetyltransferase, cytosolic 2-like	359497005	42.60	9.59	-	+	+
*01*.*07 Metabolism of vitamins*, *cofactors*, *and prosthetic groups*						
c-1-tetrahydrofolate synthase, cytoplasmic	359479954	32.70	7.12	+	-	-
*01*.*20 Secondary metabolism*						
4-coumarate—CoA ligase-like 7	225436506	59.50	9.59	+	-	-
chalcone—flavonone isomerase 2	225448801	25.10	5.13	-	-	+
Polyphenol oxidase, chloroplastic	1172587	67.30	6.28	+	-	-
**02 Energy**						
*02*.*01 Glycolysis and gluconeogenesis*						
Enolase	225455555	48.10	6.16	-	+	-
enolase 1	225441000	47.80	5.60	+	+	-
glyceraldehyde-3-phosphate dehydrogenase A, chloroplastic-like	225451685	43.10	7.76	+	-	-
glyceraldehyde-3-phosphate dehydrogenase B, chloroplastic isoform 2	225457604	47.20	7.84	+	-	-
glyceraldehyde-3-phosphate dehydrogenase, cytosolic	359491599	36.70	8.72	+	+	+
glyceraldehyde-3-phosphate dehydrogenase-like	225425884	36.70	7.77	-	+	+
protein disulfide-isomerase	225459587	55.60	4.79	-	-	+
triosephosphate isomerase, cytosolic	225449541	27.10	6.42	+	+	-
*02*.*10 Tricarboxylic-acid pathway*						
malate dehydrogenase, chloroplastic-like	225452831	43.70	9.06	+	+	+
malate dehydrogenase, cytoplasmic	225438145	35.50	6.20	+	+	-
malate dehydrogenase, mitochondrial	225443845	36.80	9.51	+	+	+
NADP-dependent malic enzyme	1708924	65.20	6.07	+	+	-
*02*.*11 Electron transport and membrane-associated energy conservation*						
enoyl-[acyl-carrier-protein] reductase [NADH], chloroplastic-like	225441423	41.60	9.42	-	-	+
*02*.*16 Fermentation*						
alcohol dehydrogenase 7	7264742	39.40	5.95	-	+	-
pyruvate decarboxylase 1	10732644	62.20	6.10	-	+	-
pyruvate decarboxylase isozyme 1	225443847	62.40	5.59	+	+	-
*02*.*30 Photosynthesis*						
chlorophyll a-b binding protein 151, chloroplastic	225447576	28.40	5.61	+	-	-
chlorophyll a-b binding protein 40, chloroplastic isoform 1	225463428	27.90	4.97	+	-	-
photosystem II 44 kDa protein	91983988	51.80	6.76	+	-	-
*02*.*45 Energy conversion and regeneration*						
ADP, ATP carrier protein 1, mitochondrial-like	225450149	42.00	10.18	+	+	-
ATP synthase subunit beta, mitochondrial-like	225456079	59.10	5.86	+	+	-
ATP synthase subunit O, mitochondrial isoform 1	225450135	27.50	10.04	-	+	-
ATPase subunit 1	224365668	55.10	5.97	+	+	-
ATP-citrate synthase alpha chain protein 2 isoform 1	225450474	46.40	5.22	+	-	-
ATP-citrate synthase beta chain protein 2	225431960	65.90	7.10	-	+	+
V-type proton ATPase subunit B 1-like	225459744	54.20	4.85	-	+	-
**11 Transcription**						
*11*.*02 RNA synthesis*						
flavoprotein wrbA isoform 1	225461209	21.70	5.80	+	-	-
**12 Protein synthesis**						
*12*.*01 Ribosome biogenesis*						
40S ribosomal protein S16	225428853	16.40	10.21	+	-	-
40S ribosomal protein S23-like	225435203	15.60	11.02	+	+	-
40S ribosomal protein S5 isoform 2	225441583	23.10	10.19	+	-	-
60S ribosomal protein L35-like	225448819	14.30	11.39	-	+	-
putative 40S ribosomal protein S5, partial	37780996	16.80	11.26	-	+	+
*12*.*04 Translation*						
elongation factor 1-alpha	225439902	49.30	9.76	+	-	-
elongation factor 1-alpha-like	225435233	49.30	9.76	-	+	-
elongation factor 2-like isoform 1	225462164	93.90	5.74	+	+	-
seryl-tRNA synthetase	225450981	51.10	6.27	+	-	-
*12*.*07 Translational control*						
eukaryotic initiation factor 4A-2	225442221	46.90	5.34	-	+	-
**14 Protein fate (folding, modification, destination)**						
*14*.*01 Protein folding and stabilization*						
97 kDa heat shock protein-like	359482944	93.30	4.99	-	+	-
heat shock cognate 70 kDa protein isoform 2	359486799	71.10	5.02	-	+	-
heat shock cognate protein 80-like	359495606	80.00	4.84	+	+	-
luminal-binding protein 5-like	359490716	73.40	4.90	+	-	-
peptidyl-prolyl cis-trans isomerase CYP20-3, chloroplastic-like isoform 2	359480227	22.50	9.63	+	+	-
peptidyl-prolyl cis-trans isomerase isoform 1	225457957	17.90	9.87	+	+	+
*14*.*07 Protein modification*						
aspartic proteinase	144228219	20.80	4.48	-	+	-
aspartic proteinase nepenthesin-2	225455876	53.10	5.64	-	-	+
dolichyl-diphosphooligosaccharide—protein glycosyltransferase subunit 2-like	359480291	75.30	5.62	-	+	-
probable acetyl-CoA acetyltransferase, cytosolic 2-like	359497005	42.60	9.59	+	-	-
*14*.*10 Assembly of protein complexes*						
coatomer subunit gamma-2-like	359475304	98.60	4.94	+	+	+
ruBisCO large subunit-binding protein subunit beta, chloroplastic	225435794	64.60	5.71	+	+	-
*14*.*13 Protein/peptide degradation*						
probable mitochondrial-processing peptidase subunit beta	225452974	58.40	6.45	+	-	-
**16 Protein with binding function or cofactor requirement (structural or catalytic)**						
*16*.*07 Metal binding*						
aconitate hydratase, cytoplasmic	225447278	107.40	7.84	-	-	+
aconitate hydratase 2, mitochondrial	225460961	110.00	6.71	+	+	-
*16*.*21 Complex cofactor/cosubstrate/vitamine binding*						
membrane steroid-binding protein 2	225470692	23.50	4.57	+	-	-
**20 Cellular transport, transport facilitation and transport routes**						
*20*.*01 Transported compounds (substrates)*						
importin subunit alpha-1	225431871	58.10	5.18	-	-	+
*20*.*03 Transport facilities*						
ABC transporter C family member 8-like	359482526	164.10	9.04	+	-	-
**32 Cell rescue, defense and virulence**						
*32*.*01 Stress response*						
catalase isozyme 1-like	359476986	56.90	6.77	+	+	-
peroxidase 4	225434381	34.00	9.56	+	-	-
*32*.*05 Disease*, *virulence and defense*						
major allergen Pru ar 1	225431840	17.30	5.10	+	-	-
**34 Interaction with the environment**						
*34*.*11 Cellular sensing and response to external stimulus*						
temperature-induced lipocalin	77744883	21.50	6.63	+	+	+
major allergen Pru av 1	225431844	17.10	5.96	+	-	-
**36 Systemic interaction with the environment**						
*36*.*20 Plant/fungal specific systemic sensing and response*						
vicilin-like antimicrobial peptides 2-1-like	359479651	63.90	7.84	-	-	+
*Vitis vinifera* Thaumatin-Like (VVTL) Proteins	410563154	21.30	4.76	+	+	-
**40 Cell fate**						
cell division cycle protein 48 homolog	225436524	89.10	5.19	+	+	+

**Fig 1:**
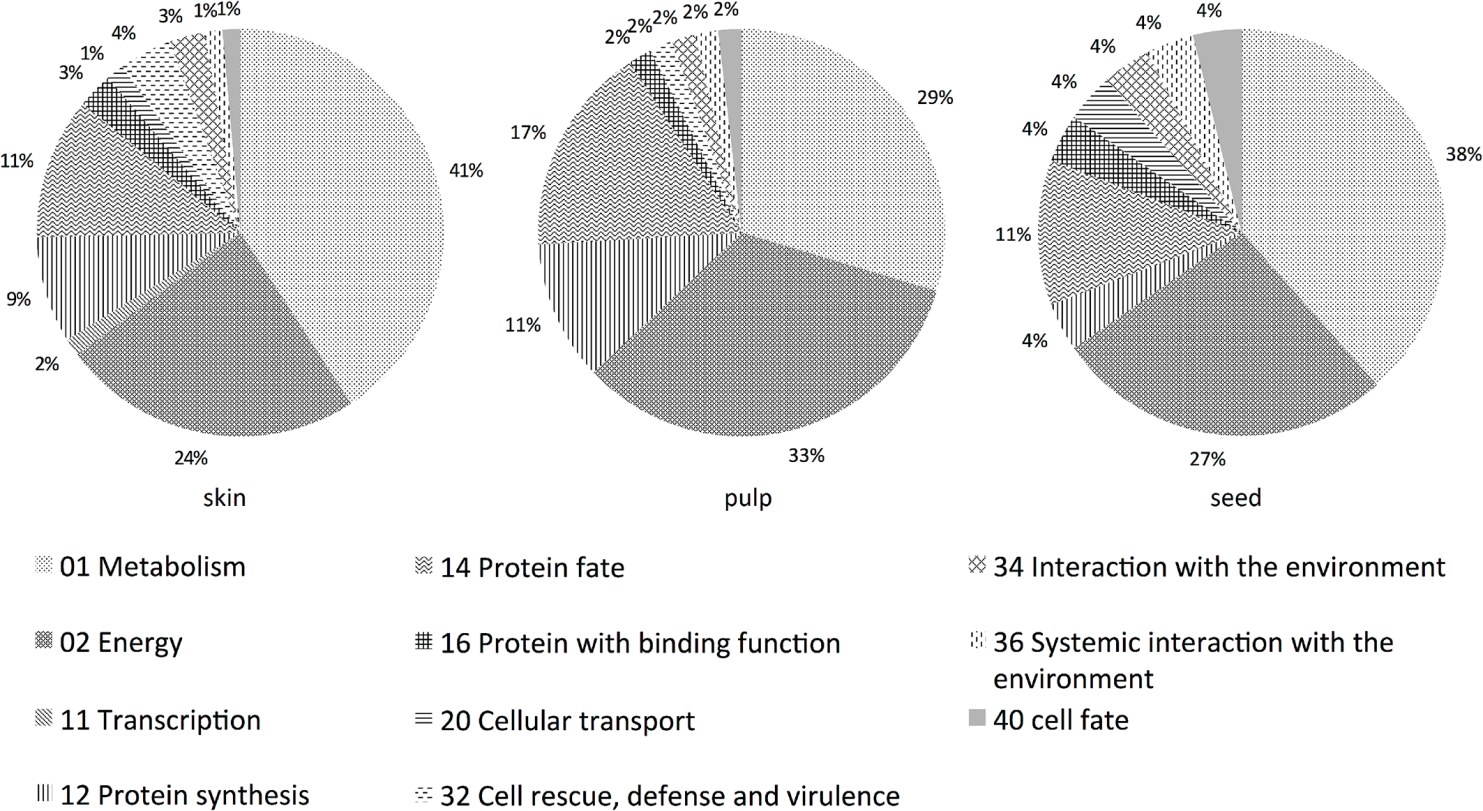
Functional distribution of the identified proteins in Sauvignon Blanc grape skin, pulp and seed.

The functional distribution of identified proteins in specific grape tissues is shown in Fig 1. Most of the proteins extracted from Sauvignon Blanc grape tissues fell into the groups labelled as metabolism (41% for skin tissues, 29% for pulp tissues, and 38% for seed tissues), energy (24% for skin tissues, 33% for pulp tissues, and 27% for seed tissues), protein fate (11% for skin tissues, 17% for pulp tissues and 11% for seed tissues) and protein synthesis (9% for skin tissues, 11% for pulp tissues and 4% for seed tissues). The high frequency of proteins involved in metabolism and energy confirmed results of previous proteomic studies [24,31]. In addition, proteins identified in grape seed were less diverse than those identified in grape skin and pulp.

**Fig 2:**
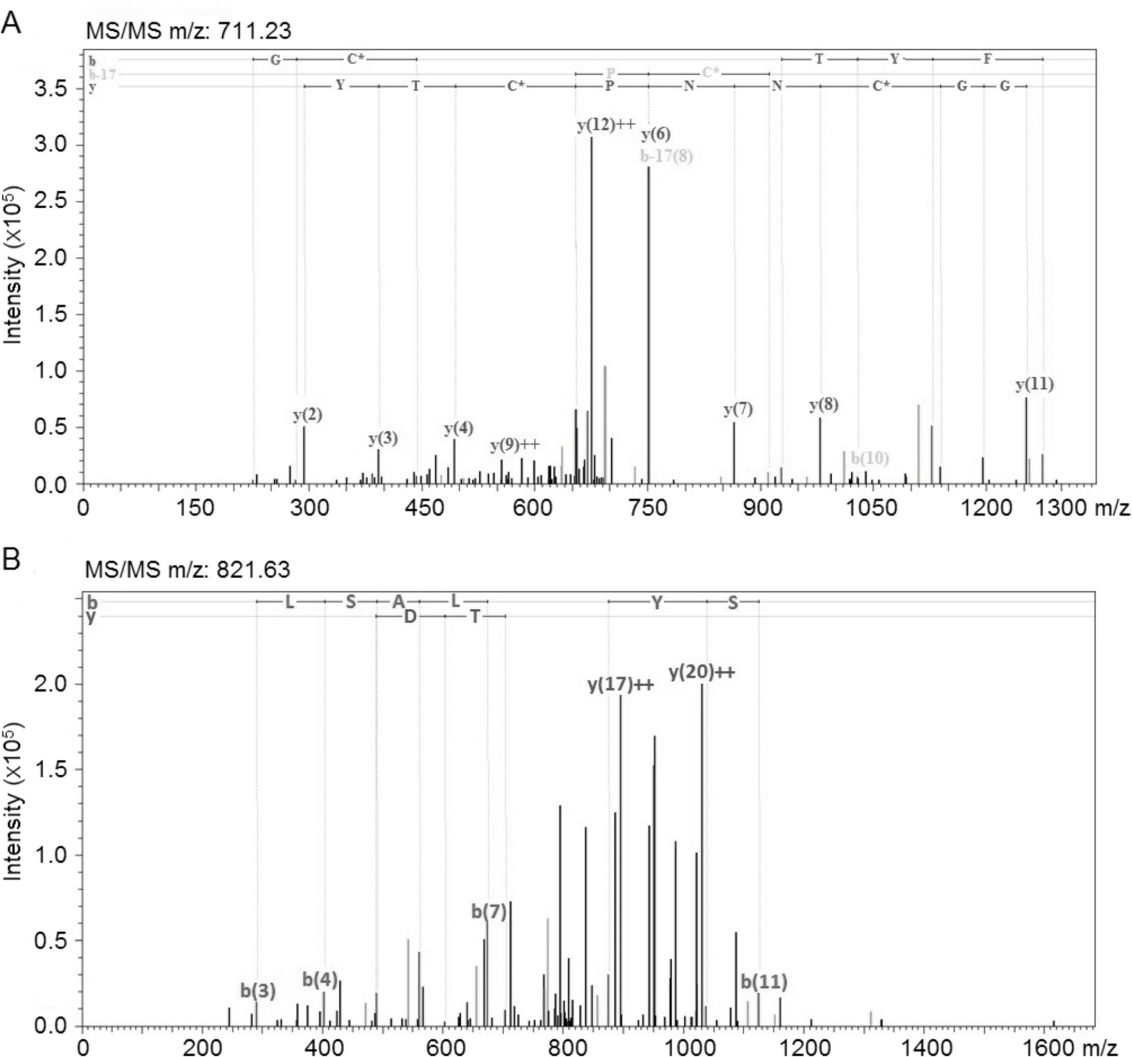
The MS/MS spectrum of 711.23 m/z (A) and 821.63 m/z (B), which identified the peptide unique to VVTL1 and chitinases from *Vitis vinifera*, respectively.

The *Vitis vinifera* thaumatin-like proteins (VVTL1), one of TLPs isoforms [32], and chitinases were identified in both grape skin and pulp but not in the seed. The MS/MS analysis of a peptide from VVTL1 and chitinase are shown in Fig 2. In this study, more than one isoform of TLPs could be present but it was not possible to identify these because of high sequence homology between the isoforms thus reducing the probability of identifying unique peptides. These results are in agreement with previous studies in which the PR proteins were observed in grape skin and pulp of other grape cultivars [14,23,33]. The observation of PR proteins in grape skin suggests that grape processing techniques which facilitate skin extraction may result in increased PR protein extraction into juice, and may affect the final protein concentration in and bentonite requirement of wine. In this study, another PR protein, β-1,3- glucanase, was detected in Sauvignon Blanc grape skin. In a recent study, Wang et al [34] also observed β-1,3- glucanase in the skin of Sangiovese and Trebbiano. These results suggest that skin extraction can contribute to protein composition in wine and the β-1,3- glucanases observed in wine are likely to be derived from grape skin.

### SDS-PAGE analysis of proteins seperated by HPLC

**Fig 3:**
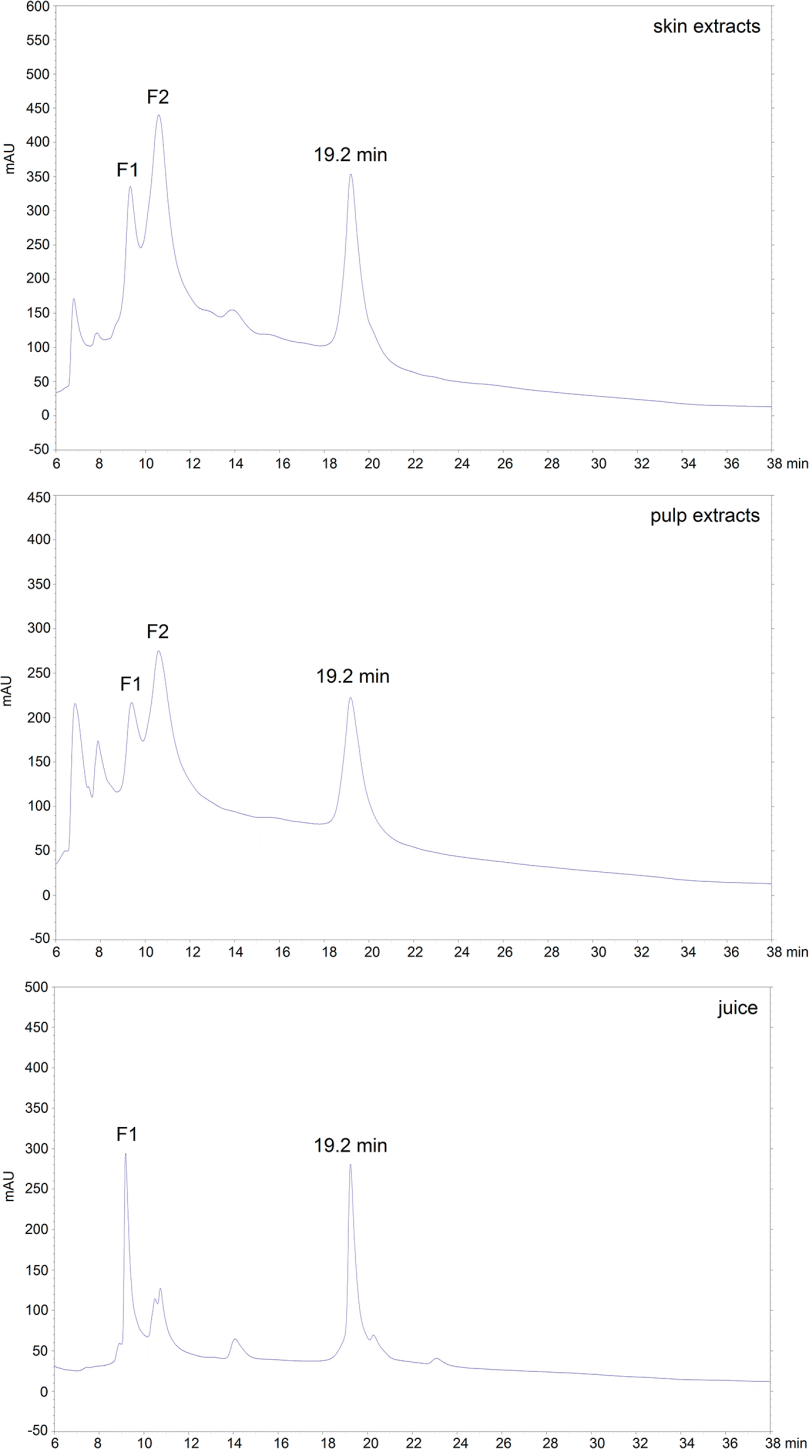
Comparison of chromatograms of protein extracts and juice from HPLC.

**Fig 4:**
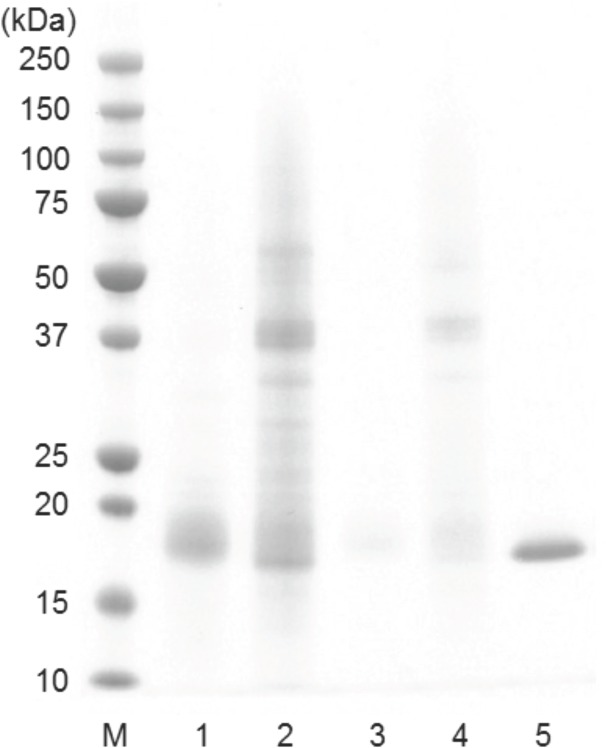
The SDS-PAGE analysis of protein fractions separated by HPLC: M, protein molecular weight marker; 1–2, F1 and F2 seperated from skin protein extracts and eluted at 9.3 min and 10.6 min respectively; 3–4, F1 and F2 seperated from pulp protein extracts and eluted at 9.3 min and 10.6 min respectively; 5, F1 (assigned to TLPs) collected from Sauvignon Blanc juice.

In order to identify and quantify the PR proteins from the protein extracts of grape tissues using HPLC, the retention times of all protein peaks from the protein extracts were compared with the retention times of purified TLPs and chitinases and with those previously reported [10,29,30]. For protein extracts from both grape skin and pulp, there were two adjacent peaks eluted at the retention time of 9.3 min (F1) and 10.6 min (F2), respectively, and there was a single peak eluted at retention time of 19.2 min (Fig 3). As described above, the fraction 1 (F1) from Sauvignon Blanc juice was assigned to TLPs and proteins eluted at 19.2 min were assigned to chitinases, and proteins eluted at 9.3 min and 10.6 min were collected and further analysed by SDS-PAGE. The analysis of these two protein fractions together with the TLPs separated from Sauvignon Blanc juice, for comparison, is shown in Fig 4. Although the molecular mass of TLPs determined by mass spectrometry was 21.3 kDa, the SDS-PAGE analysis result showed that the molecular weight of TLPs was about 18 kDa. In a previous study, Le Bourse et al. [35] also observed that the molecular weight of TLPs determined on electrophoresis gel was smaller than its theoretical value determined by mass spectrometer. The SDS-PAGE analysis showed that the main protein peak in F1 had a MW of 18 kDa which was presumably TLP. Thus, the protein fraction eluting at 9.3 min was assigned to TLP and the area of this peak was used to relatively quantify TLP in protein extracts of grape tissues. In addition, the analysis of protein fraction 2 on SDS-PAGE showed that proteins with MW of 37 kDa were observed in protein extracts from both grape skin and pulp, and these proteins were presumably glucanases which were observed in wine by Sauvage et al. [36].

### Relative quantification of PR proteins in grape skin and pulp

Relative quantification of TLPs and chitinases was carried out by comparing the area of the peaks eluted at 9.3 min and 19.2 min, respectively, against the area of a thaumatin standard. Using this method, the concentrations of TLPs and chitinases were determined in Sauvignon Blanc grape skin and pulp (Table 2). The results represented three measurements of TLPs and chitinases in specific grape tissues by HPLC. Comparison of the concentrations between two grape tissues showed that Sauvignon Blanc grape pulp contained similar concentration of TLPs and chitinases (275.1 mg/L and 248.2 mg/L, respectively). The concentrations of TLPs and chitinases determined in Sauvignon Blanc grape skin were 581.8 mg/L and 442.4 mg/L, respectively. In consideration of the weight ratio between skin and pulp in a single berry (approximately 1:8.8), the amount of these PR proteins in grape skin on per berry basis are actually much less than those in grape pulp. Furthermore, in this study the concentrations of TLPs and chitinases determined in grape skin and pulp were much higher than those reported in a previous study [37] in which the proteins were extracted by homogenizing the grape tissues in model grape juice, this is possibly because the protein extraction method used in current study was optimized for grape berries which have high sugar content and large amounts of polyphenols that can interfere the efficiency of protein extraction [25]. Furthermore, in this study, the observation of TLPs and chitinases in Sauvignon Blanc grape skin supported the conclusion that higher concentration of PR proteins in juice from mechanically harvested grapes coupled with long distance transport was likely caused by greater extraction of PR proteins from grape skin [37]. However, the concentration of PR proteins in juice is predominently determined by their concentration in grape pulp [38], the effect of skin extraction on enhancing the concentration of PR proteins in juice might be limited due to the relatively low amount of TLPs and chitinases in grape skin.

**Table 2:** Quantification of PR protein in Sauvignon Blanc grape skin and pulp (*n* = 3).

Tissue	TLPs (mg/L)*	Chitinases (mg/L)*
Skin	581.8 ± 17.2	442.4 ± 34.3
Pulp	275.1 ± 7.1	248.2 ± 14.1

* The concentration was determined using HPLC, and expressed as thaumatin equivalent.

## Conclusions

Protein profiling of New Zealand Sauvignon Blanc was carried out in this study to provide additional information on the distribution and composition of proteins in specific grape tissues. Two major soluble haze-forming PR proteins, TLPs and chitinases, were identified in both Sauvignon Blanc grape skin and pulp. Furthermore, the relative quantification of TLPs and chitinases showed that their concentration on a per berry basis in grape pulp was mucher higher than those in grape skin. These results indicate that the predominent PR proteins in grape juice are likely coming from the pulp, but the potential for extraction of PR proteins from grape skin during juicing process may consequently increase the concentration of PR proteins in juice and thus require higher bentonite addition for resultant wine stabilization.
